# Mapping reticulospinal drive across various muscles of the upper and lower extremities

**DOI:** 10.1113/EP092763

**Published:** 2025-05-05

**Authors:** Antonia Maria Eilfort, Lennart Carlson Neumann, Linard Filli

**Affiliations:** ^1^ Spinal Cord Injury Center Balgrist University Hospital Zurich Switzerland; ^2^ Neuroscience Center Zurich University of Zurich Zurich Switzerland; ^3^ Department of Health Science and Technology ETH Zurich Zurich Switzerland; ^4^ Swiss Center for Movement Analysis (SCMA) Balgrist Campus AG Zurich Switzerland

**Keywords:** corticospinal system, descending movement control, motor systems, reticulospinal motor control, reticulospinal system, StartReact

## Abstract

The reticulospinal (RS) system is a fundamental descending pathway involved in human movement control. However, the relative strength of RS projections across different muscles and its specific contributions to distinct movements are poorly understood. We systematically mapped the RS drive across a broad range of muscles in the upper and lower extremities. The RS drive was assessed in 14 muscles of 29 healthy participants using the StartReact paradigm, characterized by shortened premotor reaction times when movement initiation is paired with a loud versus moderate acoustic stimulus. Reaction times were assessed by surface EMG. RS drive was compared as follows: (1) across individual muscles; (2) between proximal and distal muscles; and (3) between flexor and extensor muscles. The RS drive was lowest in the finger abductor, with significantly reduced values in comparison to those in the shoulder flexor and extensor, the elbow flexor, hip and knee extensors, and the ankle plantar flexor. A proximal‐to‐distal gradient in RS drive was observed only in the upper extremities, mainly attributable to the low RS drive to the finger abductor. Additionally, the RS drive was greater to flexors than to extensors in the upper extremities. Conversely, the RS drive was enhanced to extensors versus flexors in the lower extremities. Our findings emphasize the presence of RS drive in all examined muscles, with no distinctive proximal‐to‐distal gradient in RS motor control. Notably, a reversed flexor–extensor bias in RS control was evident between the upper and lower extremities. These findings advance our understanding of RS motor control and might inform the development of targeted neurorehabilitation strategies.

## Abstract (highlights)

- **What is the central question of this study?**
  The reticulospinal system is a key descending motor pathway, yet its role in human movement control remains poorly understood. This study presents the first comprehensive assessment of reticulospinal drive across 14 upper and lower extremity muscles, examining proximal–distal and flexor–extensor gradients.
- **What is the main finding and its importance?**
  Reticulospinal drive is enhanced to proximal compared to distal muscles in upper extremities, with no such gradient in lower extremities. Reticulospinal drive is more pronounced to flexors in upper extremities, whereas it is strongly enhanced to extensors in lower extremities. These findings provide new insights into subcortical motor control, laying a foundation for development of neurorehabilitation strategies.

## 1 INTRODUCTION

The corticospinal (CS) and reticulospinal (RS) systems are the principal descending motor pathways that control human movements. The CS system has been investigated extensively, probably owing to the accessibility of the motor cortex for neuromodulation (e.g., by transcranial magnetic stimulation) and neural recordings (e.g., EEG). CS projections were shown to innervate specific groups of spinal interneurons and motoneurons, enabling skilled movements, such as individual finger movements (Baker et al., 2015; Lemon, 2008).

Preclinical studies emphasize the RS system as a fundamental motor pathway able to mediate basic movement control in the absence of CS commands: following bilateral pyramidotomy, non‐human primates quickly regain walking and climbing abilities but exhibit lasting impairments in dexterous hand functions, such as grasping (Lawrence & Kuypers, 1968). This suggests that although cortical control is crucial for precise goal‐oriented movements, essential aspects of corticomotor control seem to be mediated by indirect pathways, such as the cortico‐reticulospinal pathway (Asboth et al., 2018). The descending RS fibres project to interneurons and motoneurons across multiple spinal segments bilaterally (Davidson & Buford, 2006; Riddle et al., 2009), coordinating multi‐joint movements, such as locomotion, posture and reaching (Akalu et al., 2023). However, the role of the RS system in human motor control remains poorly understood. The StartReact paradigm, characterized by a shortened reaction time (RT) when movement initiation is paired with a loud acoustic stimulus (LAS), offers a way to examine RS motor control in humans (Valls‐Solé et al., 1995). Although the neural mechanisms of the StartReact effect are not fully understood, substantial evidence indicates that this effect is linked to RS motor drive, with the StartReact amplitude reflecting the relative contribution of the RS system (Nonnekes et al., 2014; Tapia et al., 2022).

The RS innervation pattern across muscle groups in humans remains insufficiently understood. Most studies on RS motor control have examined only limited numbers of muscles, often focusing on a single muscle, thus not allowing comprehensive patterns in RS drive to be unravelled. The limited research examining RS drive across multiple muscles suggests higher RS projections to proximal versus distal muscles in the upper extremities, with pronounced RS drive to shoulder and elbow muscles in comparison to finger muscles (Carlsen et al., 2009; Castellote & Kofler, 2018; Maslovat et al., 2023; McPherson et al., 2018; Mooney et al., 2023). However, these findings often rely on comparisons of RS drive between only two muscles, with the distal muscle typically represented by a finger muscle. Preclinical findings, in contrast, do not support a prominent proximal‐to‐distal gradient in RS motor control (Buford & Davidson, 2004). Electrophysiological experiments in non‐human primates have demonstrated RS drive to intrinsic finger muscles at levels comparable to proximal muscles (Riddle et al., 2009). A more consistent pattern of RS innervation appears to be a flexor bias in the upper extremities. Neurophysiological findings in non‐human primates highlight that RS drive typically facilitates flexors while suppressing extensors of the ipsilateral side (Davidson & Buford, 2006; Davidson et al., 2007). Enhanced RS projections to upper extremity flexors are further supported by RS‐driven functional recovery in monkeys with CS damage, which is more pronounced in flexors than extensors (Zaaimi et al., 2012). Additionally, stroke survivors typically exhibit enhanced muscle activity in arm and hand flexors, while extensors often remain weak (Kamper et al., 2003; McPherson et al., 2018). While these findings support a flexor bias in RS drive for the upper extremities, the pattern of RS drive across human muscles in the lower extremities (flexors vs. extensors and proximal vs. distal muscles) remains unknown.

The aim of this study was to profile RS motor drive systematically across 14 upper and lower extremity muscles to unravel RS innervation patterns in humans. To our knowledge, this is the first comprehensive mapping of the RS drive across a broad range of muscles in both extremities. The findings of this study will advance our understanding of subcortical motor control in humans and might inform rehabilitative strategies targeting the RS pathway.

## 2 MATERIALS AND METHODS

### 2.1 Participants

Thirty‐two healthy participants were recruited for this study, with eligibility restricted to participants without major orthopaedic or neurological impairments or hearing deficits. Written informed consent was obtained from all participants. The study was approved by the Ethics Committee of Canton Zurich (study identity, 2021‐00973) and was conducted according to the *Declaration of Helsinki*. Three participants were excluded owing to non‐adherence to the protocol, resulting in a final population of 29 participants for data analysis (26.17 ± 3.49 years of age, 19 females).

### 2.2 Experimental set‐up

The study consisted of two visits, spaced 14 days apart, with one visit dedicated to assessing the upper and the other the lower extremities, each composed of seven tasks. The order of visits and tasks was randomized. Participants were divided into two groups, with 19 receiving auditory stimuli via headphones (NTH‐100, Rode, Sydney, NSW, Australia) and 10 via a speaker box (Electro‐Voice, ELX200, USA) positioned 0.3 m behind them. Each visit started with a familiarization session applying loud tones, during which participants were seated comfortably with their heads facing forwards. Participants were presented with five sets of warning stimuli (WS; 92 dB, 50 ms, 500 Hz), followed by five loud acoustic stimuli (LAS; 120 dB, 50 ms, 1000 Hz). After familiarization, participants completed seven experimental blocks using the StartReact paradigm. Each block included 30 imperative stimuli [20 moderate acoustic stimuli (MAS; 82 dB, 50 ms, 1000 Hz) and 10 LAS], with the sequence randomized. MAS or LAS were preceded by a WS. Interstimulus intervals (from WS to imperative stimulus) varied between 1.5 and 3 s, and intertrial intervals (from WS to the next WS) between 6 and 10 s. The pseudo‐randomized tone application served to minimize stimulus anticipation. Participants were instructed to perform the specific movement as fast as possible following the imperative stimulus (MAS or LAS), using their dominant extremity. All participants (*n* = 29) self‐reported right‐sided dominance of their lower extremities, and 28 participants reported right‐sided dominance for their upper extremities.

### 2.3 Procedures for StartReact measurements

#### 2.3.1 Shoulder tasks

For shoulder extension and flexion tasks, participants were seated in a chair without arm rests, with the arm hanging in a neutral position. To minimize upper body movements, torsos were secured to the backrest with a Velcro strap. Participants were instructed to perform the movement as fast as possible while avoiding shoulder abduction and elbow flexion.

#### 2.3.2 Elbow tasks

For elbow extension and flexion tasks, participants were seated comfortably in a chair, with their arms resting on a table at shoulder height. The arm was positioned in a semi‐pronated position with the elbow flexed to 90° for extension tasks and 10° for flexion tasks. The starting position was marked with haptic dots on the table to ensure task consistency.

#### 2.3.3 Wrist tasks

For wrist extension and flexion tasks, participants were seated, with their arms resting on a table at 10° shoulder flexion, 90° elbow flexion and in a neutral wrist position. The arm was semi‐pronated and secured in a manipulandum, consisting of a wooden board with two fixation straps to minimize elbow movements and allow isolated wrist motion. Tactile markers indicated the initial wrist position to maintain a consistent starting point.

#### 2.3.4 Finger abduction

The arm and hand position for the finger task matched that of the wrist tasks. However, the index finger was extended and rested on an adjustable wooden cube, positioned such that the index finger remained horizontal. Fingers III–V were secured to the manipulandum with Velcro straps, enabling isolated movements of the index finger.

#### 2.3.5 Hip tasks

For hip flexion tasks, participants lay supine on a therapy table, with their arms behind their heads. Participants were instructed to flex their hips without knee flexion. For hip extension tasks, participants lay prone, with their forehead resting on the table, instructed to extend their hips without knee flexion.

#### 2.3.6 Knee tasks

For knee extension and flexion tasks, participants were seated on an elevated chair, with their legs suspended and not touching the ground, to facilitate free knee movements. The upper body was secured to the backrest, to minimize trunk movements.

#### 2.3.7 Ankle tasks

For ankle dorsal extension and plantarflexion tasks, participants sat on a therapy table, with the backrest reclined to 110°. A pillow was placed under the knees to allow slight knee flexion. The heel and foot rested in a vacuum pillow to stabilize the position, minimizing hip rotation and providing a relaxed foot position.

#### 2.3.8 Toe extension

The position for toe extension tasks was identical to that used in the ankle tasks.

### 2.4 EMG analysis

Muscle activity was recorded using surface EMG from 16 sites, including bilateral sternocleidomastoids (SCM) to assess startle responses. The remaining recorded muscles were selected as primary effectors for each respective task (Table 1). EMG was recorded using bipolar Ag–AgCl surface EMG electrodes (H124SG, Kendall). Electrode positioning has been performed according to surface electromyography for the non‐invasive assessment of muscles (SENIAM) guidelines where applicable (http://seniam.org). The 14 muscles assessed are prominent, superficial muscles that can be palpated and identified easily in young healthy participants. Additionally, electrode placement was conducted by the same examiner (A.M.E.) to promote methodological consistency. Signals were sampled at 2000 Hz and recorded with a wireless EMG system (Myon Aktos, Cometa Systems, Bareggio, Italy). EMG data were bandpass filtered (10–500 Hz) and rectified using a customized Matlab script (Matlab R2020b, Mathworks Inc., Natick, MA, USA). Acoustic stimuli and EMG signals were synchronized in Vicon Nexus (Vicon, Oxford, UK). EMG onset for target muscles was determined as EMG activity exceeding the baseline mean + 2SD, with the baseline calculated as the mean EMG activity within a 100 ms window before the imperative stimulus (LAS or MAS). To confirm EMG onset, activity had to remain above baseline + 2SD for ≥5 ms. Startle responses in the SCM were assessed in a similar manner, requiring EMG activity to exceed baseline + 1SD for ≥2 ms in a time window of 20–100 ms following MAS or LAS. Startles were defined as bilateral SCM muscle responses. During familiarization trials, a median RT for SCM activation per subject was calculated, with minimum and maximum values set as thresholds for specific SCM activation in StartReact trials. This approach was applied to exclude trials where SCM activity was detected as a co‐contraction with the task muscle.

**TABLE 1:** Overview of assessed muscles and tasks: The classification of muscles as proximal and distal, in addition to flexor and extensor muscles, is reported.

Muscles	Function and tasks	Proximal vs. distal	Extensor vs. flexor
Sternocleidomastoids (bilateral)	Startle indication	–	–
Deltoideus pars clavicularis	Shoulder flexion	Proximal	Flexor
Deltoideus pars spinalis	Shoulder extension	Proximal	Extensor
Biceps brachii	Elbow flexion	Proximal	Flexor
Triceps brachii	Elbow extension	Proximal	Extensor
Flexor carpi radialis	Wrist flexion	Distal	Flexor
Extensor digitorum	Wrist extension	Distal	Extensor
First dorsal interosseus	Finger abduction	Distal	–
Rectus femoris	Hip flexion	Proximal	Flexor
Gluteus maximus	Hip extension	Proximal	Extensor
Semitendinosus	Knee flexion	Proximal	Flexor
Vastus medialis	Knee extension	Proximal	Extensor
Tibialis anterior	Ankle dorsiflexion	Distal	Flexor
Gastrocnemius medialis	Ankle plantarflexion	Distal	Extensor
Extensor hallucis brevis	Toe extension	Distal	–

### 2.5 StartReact effect and reticulospinal gain

RTs were defined as the interval between stimulus onset and EMG onset. RTs were excluded if they were <50 ms (preventing the inclusion of anticipatory movements) or >350 ms (excluding unusually slow movements; Smith et al., 2019). Based on RT, the RS gain (RSG) was calculated as follows:

$$\mathrm{RSG}=100\;-\;\left(RT_{\mathrm{LAS}}/RT_{\mathrm{MAS}}\right)\;\times \;100$$

The RSG represents the relative shortening of RT in response to LAS compared with MAS, following an approach established previously (Baker & Perez, 2017). This relative measure of RT shortening was used to facilitate accurate inter‐muscle comparisons of RS drive across different muscles.

### 2.6 Statistics

Statistical analysis was conducted with R (v.4.4.0) and Rstudio (v.2024.04.1) using the lmer function of the lme4 package for fitting linear mixed‐effects models. The significance level was set at α = 0.05 for all tests.

To test the effect of startle responses on the RT, a model was fitted with ‘startle (present or absent)’ as a fixed effect and ‘subject’ as a random effect. To assess whether the RSG differed significantly from zero, a null model was fitted with a random intercept for the subject. To investigate the effect of stimulus application (headset vs. speaker box) on RSG, a model was fitted with ‘method (headphone or speaker box)’ as the fixed effect and ‘subject’ as a random effect. To assess the influence of task order on RSG, a model was fitted with the RSG of the first visit (comprising the first seven tasks) with ‘task order’ as a fixed effect and ‘subject’ as a random effect. A type I ANOVA was performed on the fitted model, followed by Tukey's honest significance difference (HSD) test for pairwise comparison. To assess whether the task itself affected RSG, a model was fitted with ‘task’ as a fixed effect and ‘subject’ as a random effect. A type I ANOVA was performed on this model, and Tukey's pairwise comparison was performed. This analysis was performed for the upper and lower extremities both together and separately.

Subgroup analyses were performed to address different hypotheses. To assess differences in RSG between distal and proximal muscles, the shoulder flexor and extensor, elbow flexor and extensor, hip flexor and extensor, and knee flexor and extensor were classified as proximal muscles, and the wrist flexor and extensor, finger abductor, ankle dorsiflexor and plantar flexor, and toe extensor were categorized as distal muscles (Table 1). A linear mixed‐effects model was fitted with ‘muscle type (distal/proximal)’, ‘age’ and ‘sex’ as fixed factors and ‘subjects’ as random factors. A type I ANOVA was performed on this model, with the analysis conducted on the upper and lower extremities both together and separately.

Likewise, we assessed the difference in RSG between flexors and extensors. For this, the shoulder, elbow, wrist, hip, knee flexors and the ankle dorsiflexor were categorized as flexors. Conversely, the shoulder, elbow, wrist, hip, knee extensors and the ankle plantar flexor were classified as extensors. A linear mixed‐effects model with ‘muscle type (extensor/flexor)’, ‘age’ and ‘sex’ as fixed effects and ‘subjects’ as random effects. A type I ANOVA was performed on this model, with the analysis performed on the upper and lower extremities both together and separately.

Differences in RSG between upper and lower extremity muscles were assessed by fitting a linear mixed‐effects model with ‘visit (upper or lower extremities)’, ‘age’ and ‘sex’ as fixed effects and ‘subjects’ as random effects. A type I ANOVA was performed on this model.

Additionally, to test for significant interaction effects, models were fitted with interaction terms for different factors (visit × proximal/distal, visit × extensor/flexor, proximal/distal × extensor/flexor). A type I ANOVA was performed on the interaction models to assess these effects.

## 3 RESULTS

### 3.1 Effect of startle responses on RT

The incidence of startle responses was 22.1% ± 20.1% (mean ± SD) across all familiarization trials. In StartReact trials, startle incidence was reduced to 12.9% ± 14.4% (mean ± SD), probably owing to response habituation induced by repetitive LAS application during the study protocol. Although all participants revealed startle responses during familiarization trials, nine did not show startle responses in StartReact trials. There was no difference in RT between StartReact trials with and without startles in the SCM [β = −2.38, standard error (SE) = 2.07, *t* = −1.15, *p* = 0.253, *n* = 20]. Therefore, trials with and without startle responses were combined for subsequent analyses of StartReact effects and RSG.

### 3.2 The StartReact effect

**FIGURE 1:**
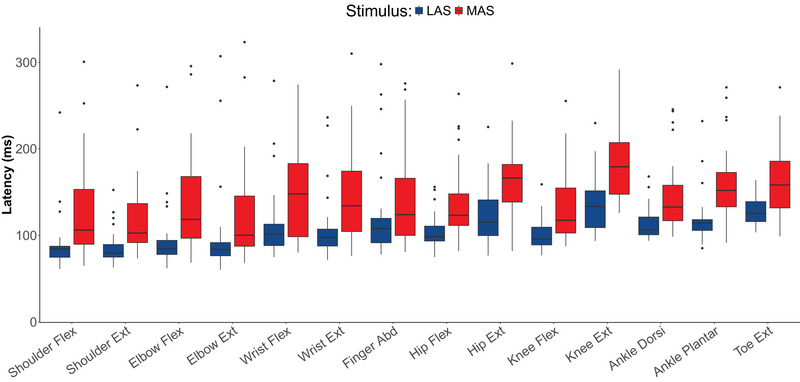
Reaction times of StartReact trials for each assessed task. Reaction times of LAS (blue) and MAS trials (in red) are displayed for all 14 tasks. Reaction times are longer following MAS than LAS, reflecting the StartReact effect. Box plots represent median values ± interquartile ranges of all participants (*n* = 29). Abbreviations: Ext, extensor; Flex, flexor; LAS, loud acoustic stimulus; MAS, moderate acoustic stimulus.

**FIGURE 2:**
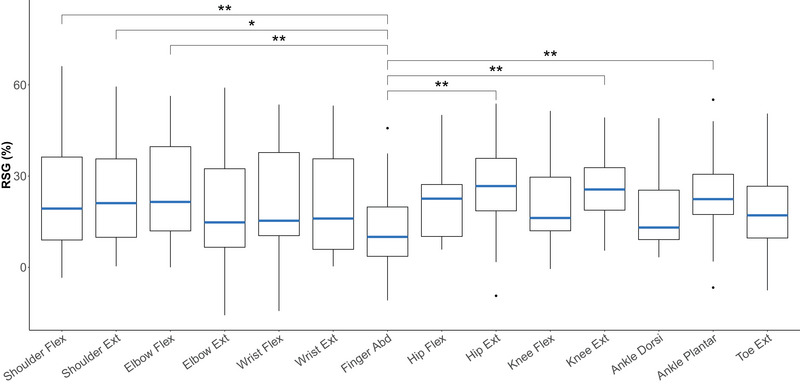
Reticulospinal gain across all upper and lower extremity tasks. Reticulospinal gain was lowest in the finger abductor, with values significantly lower than those in the shoulder flexor and extensor, elbow flexor, hip extensor, knee extensor and ankle plantar flexor. Box plots display median values with interquartile ranges for all participants (*n* = 29). Significant levels are indicated by asterisks: ^*^ *p* ≤ 0.05 and ^**^ *p* ≤ 0.01. Abbreviations: Abd, abductor; Dorsi, dorsiflexion; Ext, extensor; Flex, flexor; plantar, plantarflexion; RSG, reticulospinal gain.

We observed a significant effect of tone intensity (MAS vs. LAS) on RTs across the various tasks, reflecting the StartReact effect (Figures 1 and 2). The shortening of RTs in LAS trials compared with MAS trials manifested in RSG that was significantly different from zero (β = 21.6, SE = 1.82, *t* = 11.88, *p* < 0.0001, *n* = 29). The method for auditory stimulation (headphone vs. speaker box) did not significantly affect the RSG (β = 5.83, SE = 3.73, *t* = 1.56, *p* = 0.130, *n* = 29), indicating that the mode of tone delivery did not influence the StartReact effect. Therefore, the two groups were combined for further analysis. Additionally, the order of task performance did not significantly influence the StartReact effect: there was no effect of task order on RSG when analysing the first seven tasks of the initial experimental visit [*F*(6, 168) = 1.18, *p* = 0.321, *n* = 29]. Tukey's HSD test, which compares the different levels of the task order, revealed no significant differences between any of the levels.

### 3.3 Differences in RS drive across individual muscles of the upper and lower extremities

RSG differed significantly across muscles [*F*(13, 364) = 2.95, *p* = 0.000412, *n* = 29], indicating variations in RS drive across the different muscles. RSG was lowest in the finger abductor, and pairwise comparisons showed that the finger abductor was the only muscle with significant differences in RSG compared with other muscles (Figure 1). Notably, significant differences in RSG were observed between the finger abductor and the shoulder flexor (mean difference ± SE = −12.43% ± 3.2%, *p* = 0.00819), shoulder extensor (mean difference ± SE = –11.22% ± 3.2%, *p* = 0.0305), elbow flexor (mean difference ± SE = −13.94% ± 3.2%, *p* = 0.00116), hip extensor (mean difference ± SE = 12.8% ± 3.2%, *p* = 0.00501), knee extensor (mean difference ± SE = 14.41% ± 3.2%, *p* = 0.000604) and ankle plantar flexor (mean difference ± SE = 11.75% ± 3.2%, *p* = 0.0174).

When analysing only muscles of the upper extremities, RSG differed significantly across muscles [*F*(6, 168) = 5.22, *p* < 0.0001, *n* = 29]. Again, the finger abductor revealed the lowest RSG, which was significantly lower than RS drive to the shoulder flexor (mean difference ± SE = −12.43% ± 2.94%, *p* < 0.000442), shoulder extensor (mean difference ± SE = −11.22% ± 2.94%, *p* = 0.00244), elbow flexor (mean difference ± SE = −13.94% ± 2.94%, *p* < 0.0001), wrist flexor (mean difference ± SE = –10.43% ± 2.94%, *p* = 0.00697) and wrist extensor (mean difference ± SE = −9.68% ± 2.94%, *p* = 0.0170).

When analysing only lower extremity muscles, RSG also differed across muscles [*F*(6, 168) = 2.43, *p* = 0.0280, *n* = 29]. However, no significant differences in RSG were found between individual lower extremity muscles in the pairwise comparison.

### 3.4 RS drive to proximal vs. distal muscles of the upper and lower extremity

**FIGURE 3:**
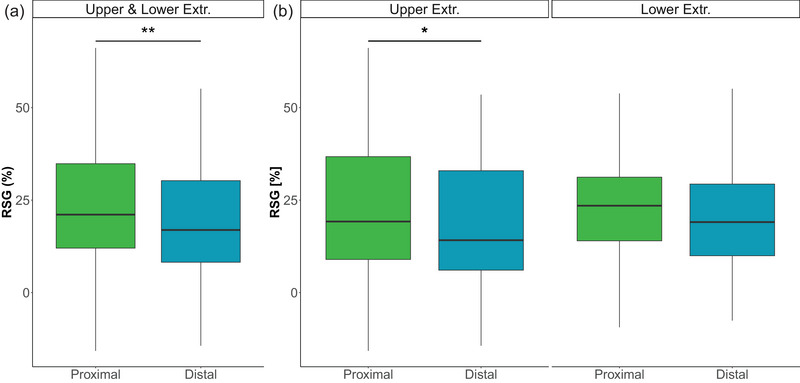
Reticulospinal drive to proximal and distal muscles. Reticulospinal gain is depicted across all muscles (a) and for muscles of the upper and lower extremities separately (b). Proximal muscles (in green) were defined as shoulder and elbow muscles (upper extremity) and as hip and knee muscles (lower extremity). Distal muscles (in blue) were defined as wrist and finger muscles (upper extremity) and as ankle and toe muscles (lower extremities). Reticulospinal gain was higher in proximal than distal muscles across all muscles (a) and in upper extremity muscles (b). Box plots display median values with interquartile ranges for all participants (*n* = 29). Significant levels are indicated by asterisks: ^*^ *p* ≤ 0.05 and ^**^ *p* ≤ 0.01. Abbreviations: Extr., extremity; RSG, reticulospinal gain.

Proximal muscles exhibited a higher RS drive than distal muscles when analysing both the upper and lower extremities (Figure 3a). This difference was supported by a significant main effect of proximal (i.e., deltoideus pars clavicularis, deltoideus pars spinalis, biceps brachii, triceps brachii, rectus femoris, gluteus maximus, semitendinosus and vastus medialis) versus distal muscles (i.e., flexor carpi radialis, extensor digitorum, first dorsal interosseus, tibialis anterior, gastrocnemius medialis and extensor hallucis brevis) on RS gain (β = 3.55, SE = 1.25, *t* = 2.84, *p* = 0.00481, *n* = 29).

A similar gradient in RS drive was observed when analysing upper extremity muscles only, with proximal muscles showing enhanced RS drive compared with distal muscles (β = 4.14, SE = 1.67, *t* = 2.48, *p* = 0.0142, *n* = 29; Figure 3b). Interestingly, when the finger abductor was excluded from the analysis, the significant difference in RSG between proximal and distal muscles disappeared (β = 0.79, SE = 1.73, *t* = 0.46, *p* = 0.649, *n* = 29), suggesting that the lower RS drive to the intrinsic finger muscle is largely responsible for the observed difference.

In contrast, the RS drive was not different between proximal versus distal muscles in the lower extremities (β = 2.9559, SE = 1.56, *t* = 1.895, *p* = 0.0597, *n* = 29; Figure 3b). Additionally, there was no significant difference in RSG between flexors and extensors within either proximal or distal muscles, as indicated by the non‐significant interaction effect [proximal/distal × extensor/flexor: *F*(1, 316) = 0.67, *p* = 0.415, *n* = 29]. Furthermore, no difference in RSG was observed between the upper and lower extremities when considering proximal and distal muscles [proximal/distal × visit (upper or lower extremities): *F*(1, 374) = 0.22, *p* = 0.636, *n* = 29].

### 3.5 RS drive to flexor vs. extensor muscles of the upper and lower extremity

**FIGURE 4:**
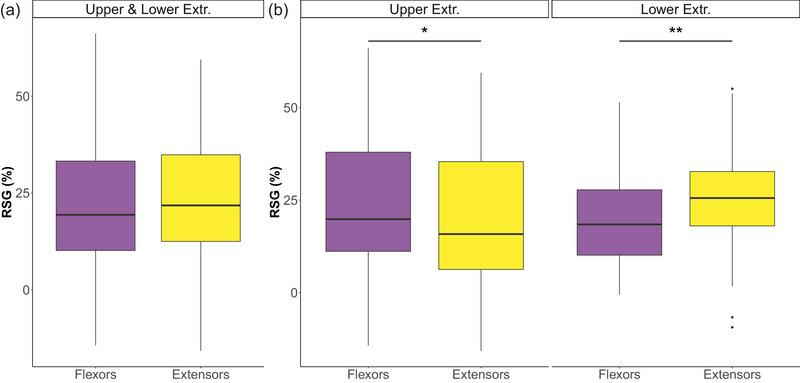
Reticulospinal drive to flexor and extensor muscles. Reticulospinal gain is depicted across all muscles (a) and for muscles of the upper and lower extremities separately (b). Flexor muscles (in purple) were defined as shoulder, elbow, wrist, hip and knee flexors, in addition to the ankle dorsiflexor. Extensor muscles (in yellow) were defined as shoulder, elbow, wrist, hip and knee extensors, in addition to the ankle plantarflexor. Reticulospinal drive was stronger to flexor than extensor muscles in the upper extremities. This pattern was inverted for the lower extremities, where reticulospinal drive was higher in extensors than flexors. Box plots display median values with interquartile ranges for all participants (*n* = 29). Significant levels are indicated by asterisks: ^*^ *p* ≤ 0.05 and ^**^ *p* ≤ 0.01. Abbreviations: Extr., extremity; RSG, reticulospinal gain.

There was no overall difference in RS drive to flexors versus extensors when combining all flexor and extensor muscles of the upper and lower extremities (β = −0.14, SE = 1.27, *t* = −0.11, *p* = 0.912, *n* = 29; Figure 4a). However, the RS drive to flexors and extensors differed considerably between the upper and lower extremities, as indicated by a significant interaction effect [extensor/flexor × visit (upper or lower extremities): *F*(1, 316) = 10.49, *p* = 0.00133, *n* = 29]. Specifically, we observed enhanced RSG in flexors compared with extensors in the upper extremities (β = 3.37, SE = 1.61, *t* = 2.09, *p* = 0.0382, *n* = 29; Figure 4b). In contrast, the RS drive was substantially higher in extensor compared with flexor muscles in the lower extremities (β = −4.89, SE = 1.55, *t* = −3.16, *p* = 0.00194, *n* = 29; Figure 4b).

### 3.6 RS drive to upper vs. lower extremity muscles

No differences in RSG were observed between muscles of the upper and lower extremities (β = −0.9, SE = 1.25, *t* = −0.72, *p* = 0.471, *n* = 29). However, as mentioned above, notable differences in RS innervation patterns were observed between upper and lower extremity muscles: a proximal‐to‐distal gradient in RS drive was present only in the upper extremities. Additionally, while the RS drive exhibited a flexor bias in the upper extremities, an extensor (or anti‐gravity) bias in RS projections was observed for the lower extremity muscles.

## 4 DISCUSSION

In this study, we systematically assessed RS motor drive across 14 muscles in the upper and lower extremities using the StartReactparadigm. RS drive was apparent in all assessed muscles, with the finger abductor exhibiting the lowest levels. Overall, the RS drive was more pronounced to proximal than distal muscles, although this gradient was caused primarily by poor RS projections to the finger abductor. Additionally, RS innervation revealed a distinct flexor‐extensor bias, with enhanced RS drive to flexors in the upper extremities and to extensors in the lower extremities. These findings provide new insights into RS innervation patterns in humans.

### 4.1 Mapping RS drive across individual muscles

The RS drive was observed across all examined muscles, including the finger abductor, although its values were significantly lower than those in the shoulder, elbow, hip, knee and ankle muscles. The presence of residual RS drive in intrinsic hand muscles agrees with neurophysiological findings in non‐human primates, which show mono‐ and disynaptic excitatory RS connections (Riddle et al., 2009). Likewise, human studies have reported auditory startle responses (Brown, 1994) and transcranial magnetic stimulation‐induced ipsilateral motor‐evoked potentials (Mooney et al., 2023), both reflecting RS drive, in the first dorsal interosseous. Supporting evidence for RS drive to finger muscles comes from research using the StartReact paradigm (Baker & Perez, 2017; Castellote & Kofler, 2018; Honeycutt et al., 2013). These studies highlight that RS contributions to distal finger movements are task dependent, increasing during tasks that engage the fingers as part of hand actions (Honeycutt et al., 2013) or during simultaneous activation of proximal muscles (Castellote & Kofler, 2018; Honeycutt et al., 2013). In contrast, the RS drive is low (Baker & Perez, 2017; Kirkpatrick et al., 2018) or absent (Carlsen et al., 2009; Maslovat et al., 2021) during individual finger movements that are controlled primarily by corticomotoneuronal connections. Aside from the reduced RS drive to the finger abductor, no significant differences in RS innervation were noted between other muscles, consistent with the broad, complex projection pattern of the RS system (Drew & Rossignol, 1990; Matsuyama et al., 1997). This divergent projection pattern might facilitate coordinated movements, such as locomotion, posture and bimanual coordination (Dietz et al., 2024; Dyson et al., 2014).

### 4.2 RS drive to proximal vs. distal muscles

RS drive was more pronounced to proximal than distal muscles across both upper and lower extremities. This supports previous reports suggesting that the RS system mainly controls axial and proximal muscles during coordinated multi‐joint movements (Grillner, 1997; Mori, 1987; Schepens & Drew, 2004), whereas dexterous hand and finger movements are governed by the CS pathway (Carlsen et al., 2009). However, the proximal‐to‐distal gradient in RS innervation in our study was driven primarily by the low RS drive to the finger abductor. When the finger abductor was excluded from analysis, the proximal‐to‐distal gradient disappeared both across all muscles and across the upper extremity muscles. Previous studies exploring RS drive to proximal and distal human muscles used only the first dorsal interosseous as a distal muscle (Carlsen et al., 2009; Castellote & Kofler, 2018; Mooney et al., 2023), thereby contributing to the oversimplified view of a distinct proximal‐to‐distal gradient in RS motor control.

Indeed, preclinical evidence challenges the notion of a proximal‐to‐distal gradient in RS drive. Experiments in non‐human primates showed that RS drive to finger and wrist motoneurons was comparable to that of shoulder and elbow muscles (Davidson & Buford, 2006; Davidson et al., 2007; Riddle et al., 2009). Notably, CS inputs to intrinsic hand muscles were about four times stronger than RS inputs, highlighting that although RS contributions to finger movements are present, they are relatively weak. Interestingly, CS projections to upper extremity muscles exhibit a proximal‐to‐distal gradient, with stronger innervation of hand and forearm muscles compared with more proximal muscles (Fritz et al., 1985; Porter & Lemon, 1993; Riddle et al., 2009). Selective ablation of CS projections in non‐human primates causes severe deficits in dexterous motor control, whereas gross motor functions, such as climbing and locomotion, recover almost completely (Hepp‐Reymond et al., 1974; Lawrence & Kuypers, 1968; Zaaimi et al., 2012). Thus, the assumption of a proximal‐to‐distal gradient in RS innervation might be shaped by insights from CS motor physiology and an oversimplified view that the two main descending motor systems operate independently. Substantial evidence supports their coordinated action in most movements (Dean & Baker, 2017; Honeycutt et al., 2013). In the lower extremities, our findings do not suggest a proximal‐to‐distal gradient in the RS drive.

### 4.3 RS drive is pronounced to upper extremity flexors and lower extremity extensors

RS innervation patterns of flexors and extensors differed between the upper and lower extremities. In the upper extremity, the RS drive was more pronounced to flexors than to extensors, consistent with findings in primates demonstrating that the RS system facilitates flexors of the shoulder and arm, while suppressing the respective antagonistic extensors on the ipsilateral side during a reaching task. A crossed pattern was reported in the contralateral arm, with the RS drive suppressing flexors and facilitating extensors (Davidson et al., 2007). This flexor bias in the upper extremity is also supported by research in able‐bodied humans, where cortico‐reticulospinal drive, as assessed by transcranial magnetic stimulation‐induced ipsilateral motor‐evoked potentials was more pronounced in flexors (i.e., biceps brachii and flexor carpi radialis) than in extensors (i.e., triceps brachii and extensor carpi radialis) (Mooney et al., 2023). Enhanced RS drive to flexors aligns with motor recovery patterns in non‐human primates with focal CS lesions and human stroke survivors, where neuroplasticity results in increased RS drive to forearm flexors but not extensors, accompanied by greater recovery of flexor muscles (Kamper et al., 2003; Zaaimi et al., 2012). Likewise, in patients with incomplete cervical spinal cord injury, the RS drive is enhanced to elbow flexors but not extensors, correlating with better recovery of elbow flexion (Sangari & Perez, 2020). Therefore, the findings of the present study confirm the flexor bias in RS control of upper extremity muscles.

RS motor control in lower extremity muscles is poorly understood. Our findings reveal an inverted RS innervation pattern in lower versus upper extremity muscles. RS drive is stronger to extensors (anti‐gravity muscles) than to flexors at the hip, knee and ankle; an observation not previously reported. Pronounced RS drive to anti‐gravity muscles agrees with the relevant role of the RS system in posture and locomotion (Juvin et al., 2016; Matsuyama & Drew, 2000; Schepens & Drew, 2004). Additionally, this pattern might be linked to the prevalence of abnormal extensor synergies and spasticity, which are more abundant in anti‐gravity muscles (Brown, 1994).

### 4.4 Implications for neural repair and motor recovery

Growing evidence highlights the significant capacity of the RS system for neuroplastic adaptations and its crucial role in functional recovery after CNS injury. Preclinical findings highlight the remarkable potential of the RS system for neuroplasticity after experimental spinal cord injury (Filli et al., 2014; Kathe et al., 2022; Zörner et al., 2014) and CS lesions (Zaaimi et al., 2012). In humans, RS plasticity is linked to functional recovery after spinal cord injury (Baker & Perez, 2017; Eilfort et al., 2024), but its role in stroke recovery appears more complex, probably depending on the extent of cortical damage. Notably, both maladaptive changes (e.g., abnormal muscle tone, spasticity, movement synergies; Arya et al., 2016; Miller & Dewald, 2012) and functional recovery patterns (Choudhury et al., 2020; Taga et al., 2024) that have been associated with RS plasticity align with the RS projection patterns identified in the present study. Flexor synergies dominate the upper extremities, whereas extensor synergies are more common in the lower extremities (Pundik et al., 2019). Additionally, recovery is greater in upper extremity flexors than in extensors, consistent with the flexor bias of RS projections. However, the mechanisms underlying the described changes after CNS injury are not fully understood and are likely to involve additional neural structures beyond the RS system. Enhanced knowledge of RS motor physiology might lead to better understanding and treatment of the pathophysiological processes occurring after spinal cord injury and stroke.

### 4.5 Limitations

Although this study provides a comprehensive overview of RS projection patterns across various muscles, the results are limited to single‐joint movements and might not translate fully to the more complex, multi‐joint movements common in daily activities, where RS drive to specific muscles could differ (Castellote & Kofler, 2018). Nonetheless, profiling RS motor control through single‐joint movements provides a crucial foundation for understanding the fundamental RS projection patterns in humans.

## 5 CONCLUSION

Our findings emphasize a widespread, divergent pattern of RS projections, resulting in RS drive apparent in all investigated muscles. Although a distinct proximal‐to‐distal gradient in RS motor control was not evident, the finger abductor received the lowest RS drive. A contrasting pattern in RS projections was observed between flexor and extensor muscles in the upper and lower extremities, in that the RS drive was enhanced to flexors compared with extensors in the upper extremities, whereas RS projections were substantially stronger to extensor muscles in the lower extremities. These results suggest that the pattern of RS innervation is more intricate than frequently portrayed in literature. Our findings deepen the limited knowledge of RS motor control and might guide rehabilitative approaches aimed at leveraging this key motor system in neurological conditions such as stroke or spinal cord injury.

## AUTHOR CONTRIBUTIONS

All experiments were performed at the Spinal Cord Injury Center of the Balgrist University Hospital and at the Swiss Center for Movement Analysis, Balgrist Campus, Zurich, Switzerland. Conception or design of the work: Antonia Maria Eilfort, Lennart Carlson Neumann and Linard Filli. Acquisition of data: Antonia Maria Eilfort, Lennart Carlson Neumann and Linard Filli. Analysis of data: Antonia Maria Eilfort. Interpretation of data: Antonia Maria Eilfort and Linard Filli. Drafting the work and revising it: Antonia Maria Eilfort, Lennart Carlson Neumann and Linard Filli. All authors have read and approved the final version of this manuscript and agree to be accountable for all aspects of the work in ensuring that questions related to the accuracy or integrity of any part of the work are appropriately investigated and resolved. All persons designated as authors qualify for authorship, and all those who qualify for authorship are listed.

## FUNDING

The study was supported by the Swiss National Science Foundation (32003B_208110) and the Balgrist Foundation (2021‐079).

## CONFLICT OF INTEREST

None declared.

## Data Availability

All data of this study are included in this manuscript (Table and Figures). Raw data may be shared upon request.
